# Supplementary figures and images for: Sickness absence and disability pension trajectories among individuals on sickness absence due to stress-related disorders. Two prospective population-based cohorts with 13-month follow-up
Source: PLoS One. 2024 Dec 13;19(12):e0315706. doi: 10.1371/journal.pone.0315706 (PMC11643301; doi:10.1371/journal.pone.0315706)

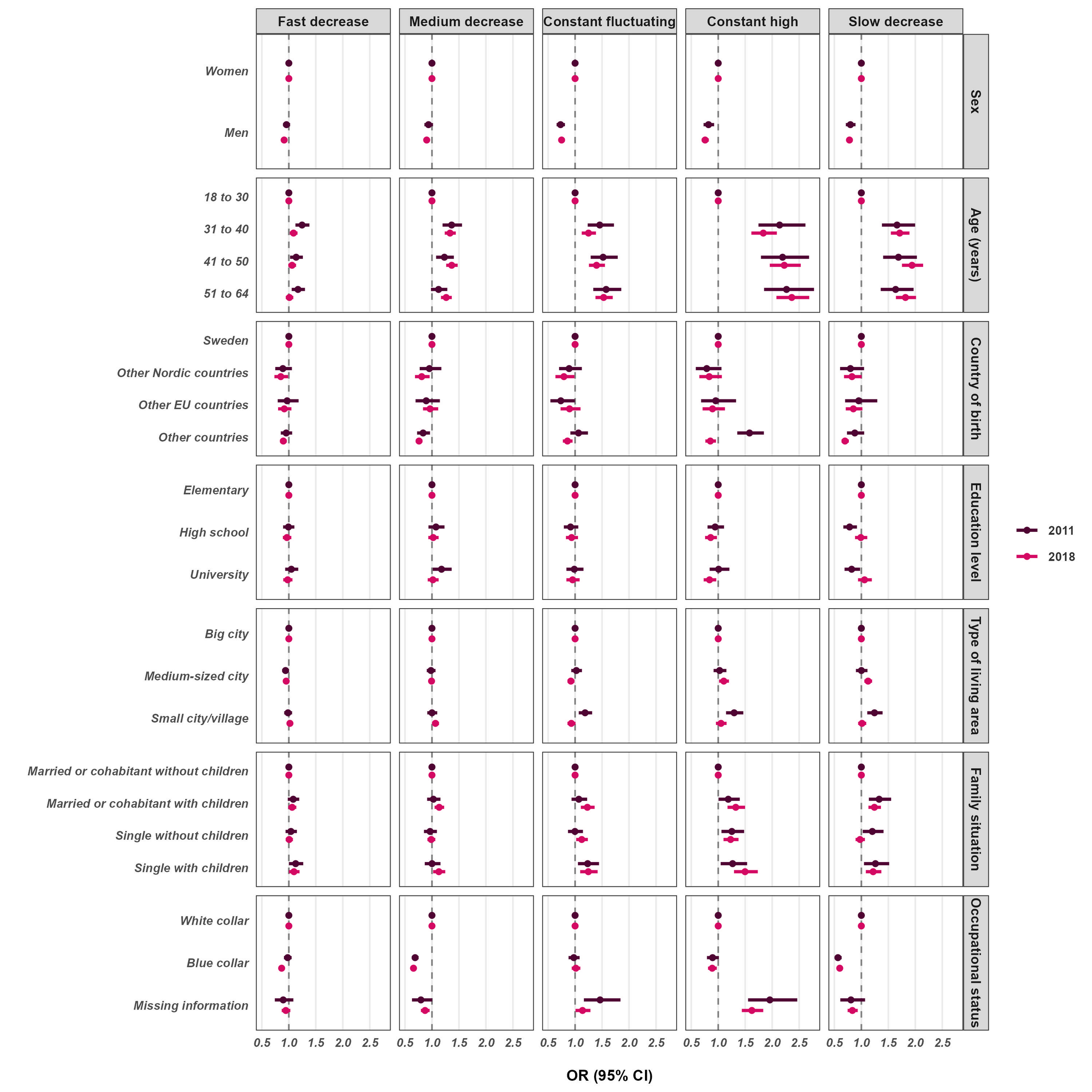

Supplement: S1 Fig — (TIF) [file pone.0315706.s001.tif]

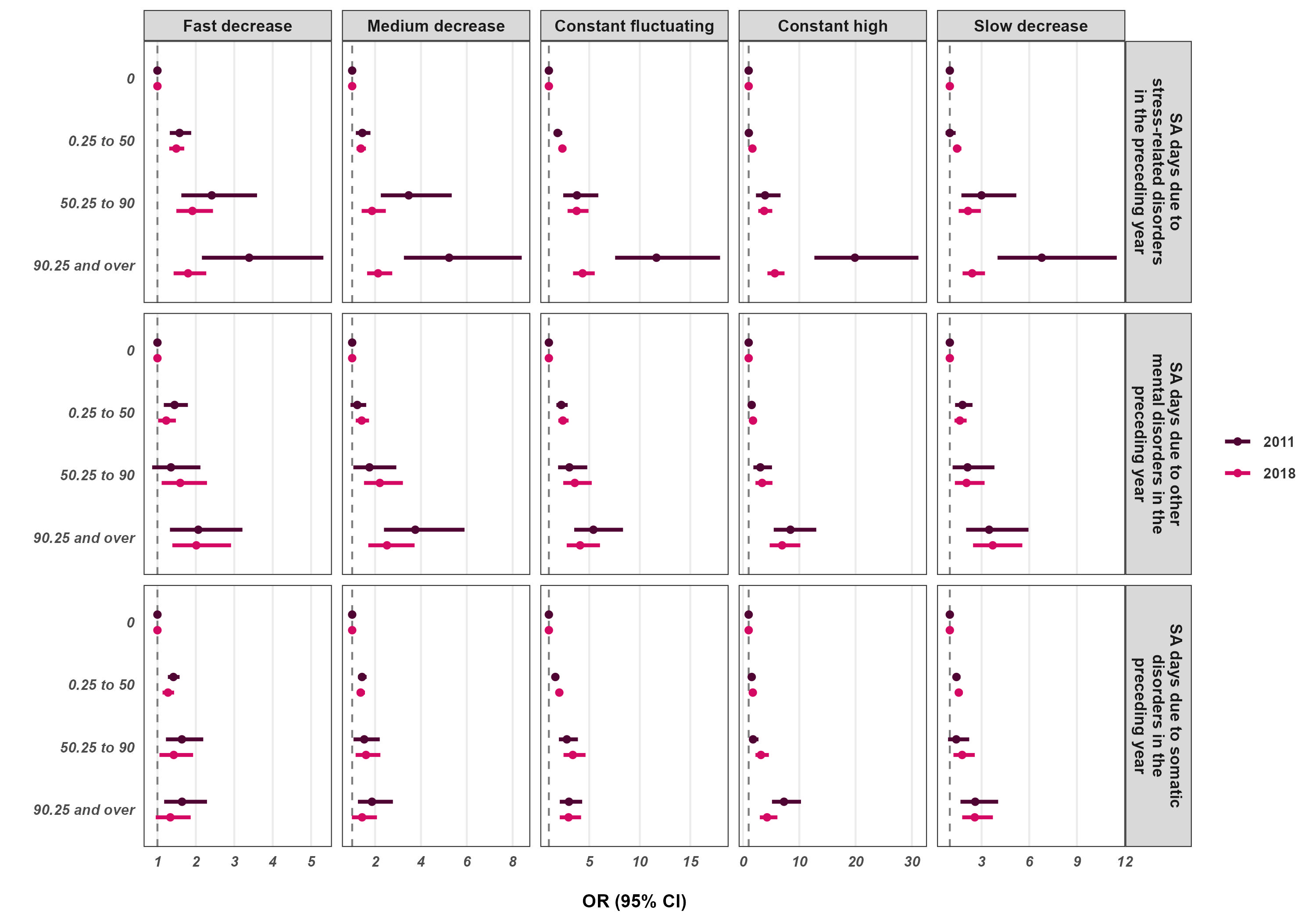

Supplement: S2 Fig — (JPEG) [file pone.0315706.s002.jpeg]

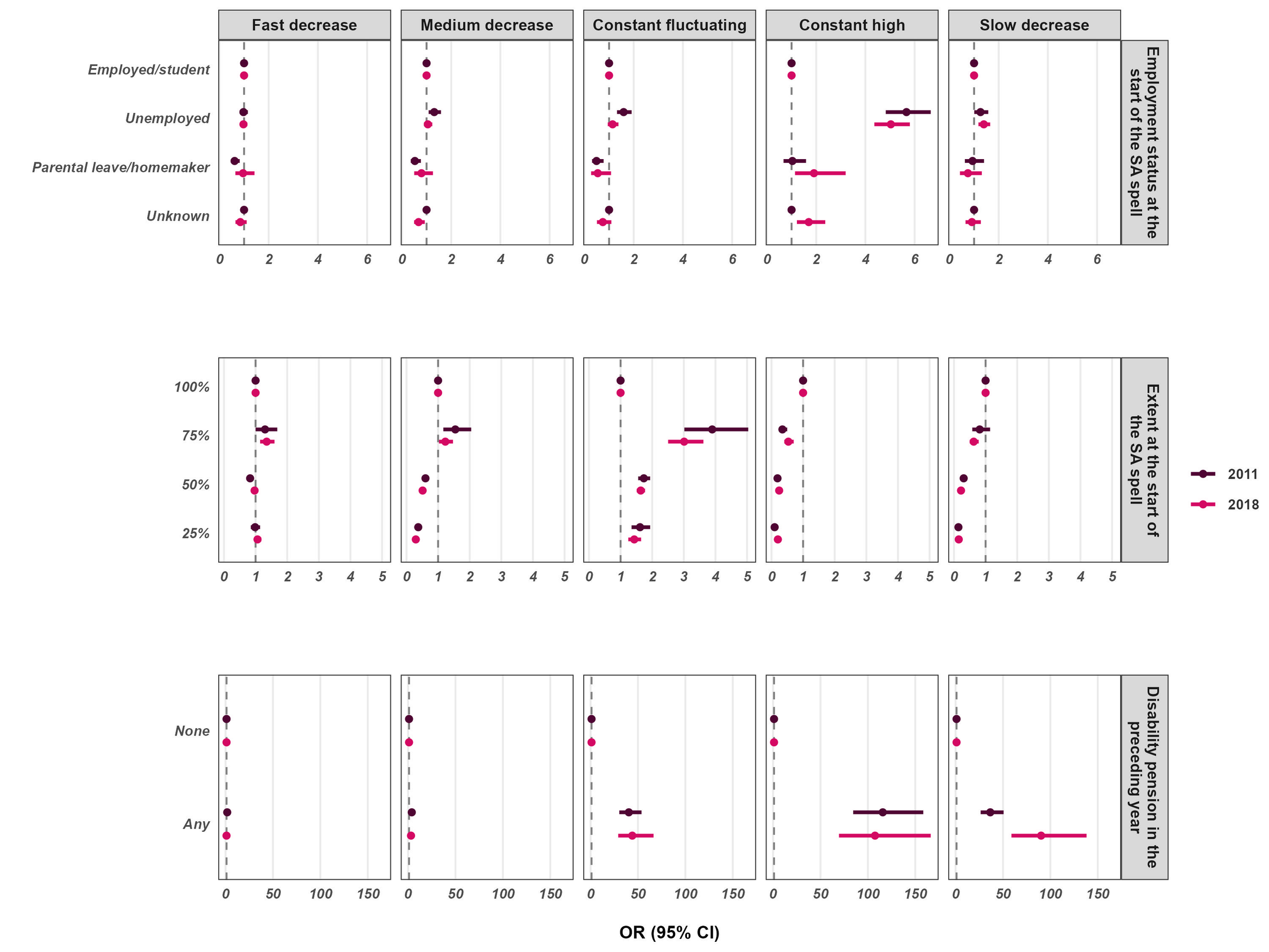

Supplement: S3 Fig — (TIF) [file pone.0315706.s003.tif]

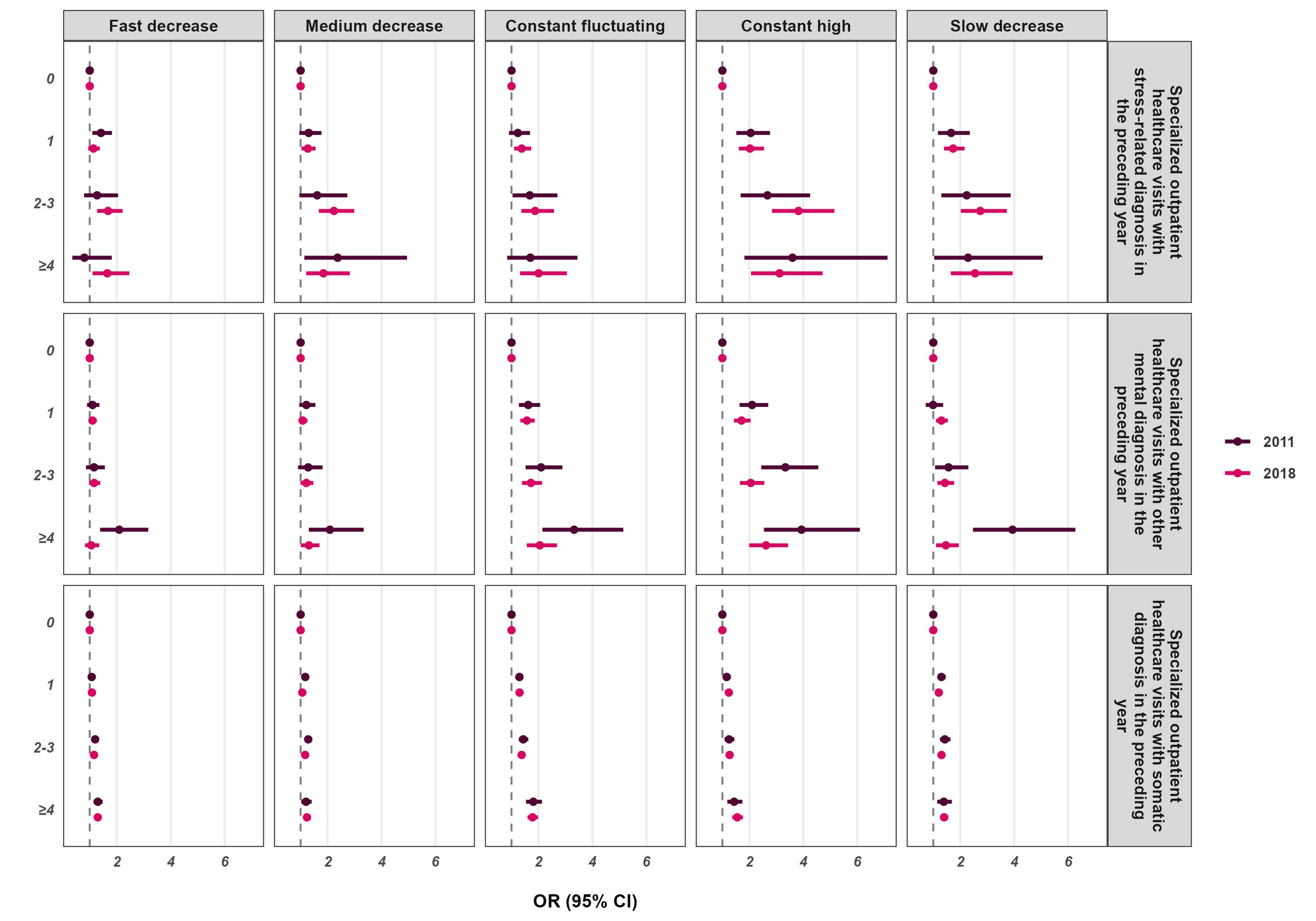

Supplement: S4 Fig — (TIF) [file pone.0315706.s004.tif]

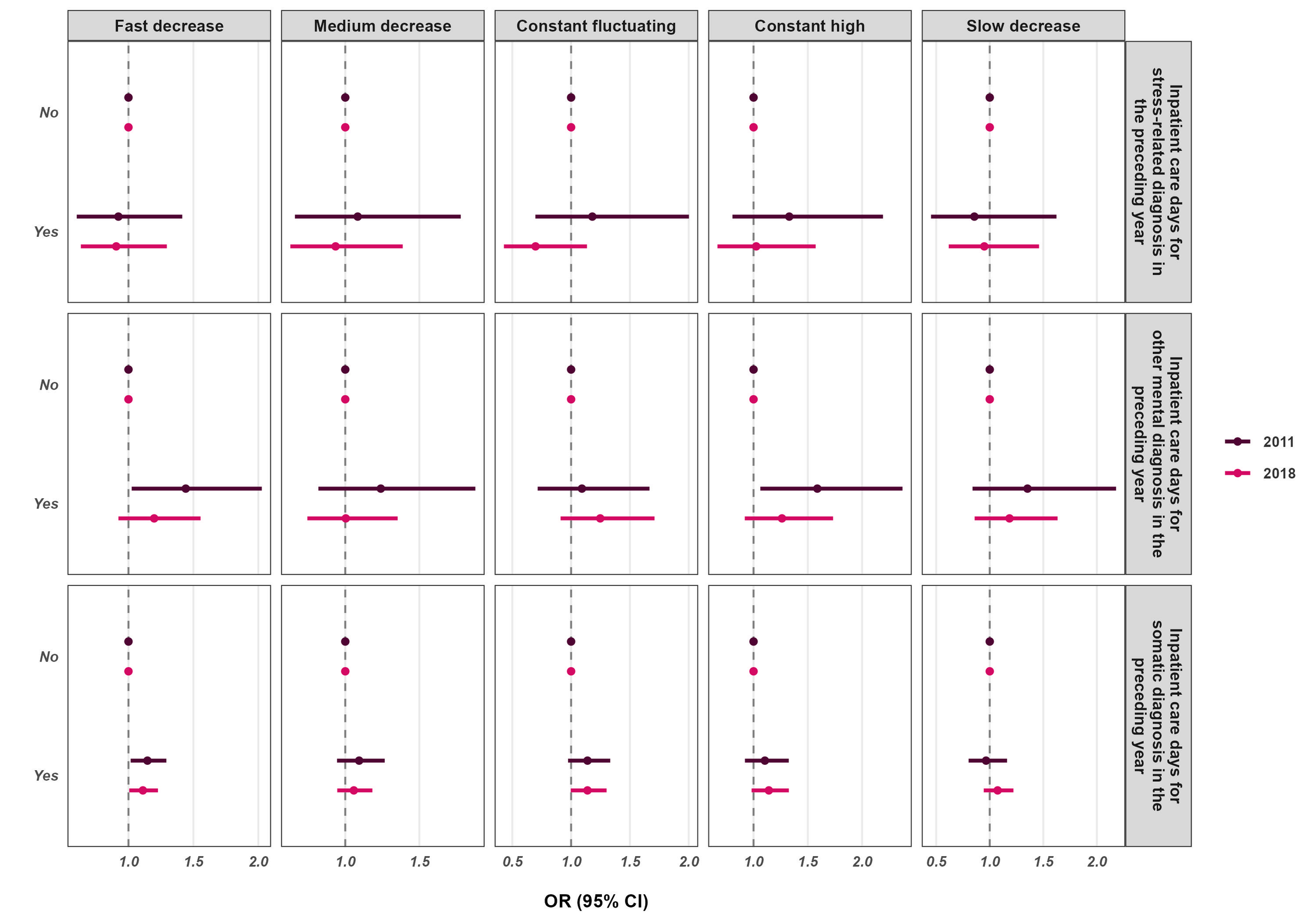

Supplement: S5 Fig — (TIF) [file pone.0315706.s005.tif]
