# Supplementary material for: Sickness absence and disability pension trajectories among individuals on sickness absence due to stress-related disorders. Two prospective population-based cohorts with 13-month follow-up
Source: PLoS One. 2024 Dec 13;19(12):e0315706. doi: 10.1371/journal.pone.0315706 (PMC11643301; doi:10.1371/journal.pone.0315706)
Supplement: S1 Table — (DOCX) [file pone.0315706.s006.docx]

**Supplement table 1.**

Distributions of sociodemographic characteristics in each trajectory group of monthly sickness absence and disability pension days among the individuals with a new sickness absence spell due to stress-related disorders.

|  | **Cohort-2011** | | | | | | **Cohort-2018** | | | | | |
| --- | --- | --- | --- | --- | --- | --- | --- | --- | --- | --- | --- | --- |
|  | **Steep drop** | **Constant fluctuating** | **Fast decrease** | **Medium decrease** | **Slow decrease** | **Constant high** | **Steep drop** | **Constant fluctuating** | **Fast decrease** | **Medium decrease** | **Slow decrease** | **Constant high** |
|  | **(N=11 641)** | **(N=3645)** | **(N=7899)** | **(N=4260)** | **(N=2379)** | **(N=2593)** | **(N=20 076)** | **(N=5682)** | **(N=16 724)** | **(N=11 890)** | **(N=7100)** | **(N=4039)** |
| **Sex** |  |  |  |  |  |  |  |  |  |  |  |  |
| Women | 8676 (74.5%) | 3023 (82.9%) | 5998 (75.9%) | 3258 (76.5%) | 1887 (79.3%) | 1968 (75.9%) | 14732 (73.4%) | 4585 (80.7%) | 12695 (75.9%) | 9027 (75.9%) | 5614 (79.1%) | 3172 (78.5%) |
| Men | 2965 (25.5%) | 622 (17.1%) | 1901 (24.1%) | 1002 (23.5%) | 492 (20.7%) | 625 (24.1%) | 5344 (26.6%) | 1097 (19.3%) | 4029 (24.1%) | 2863 (24.1%) | 1486 (20.9%) | 867 (21.5%) |
| **Age** |  |  |  |  |  |  |  |  |  |  |  |  |
| 18-30 | 1684 (14.5%) | 261 (7.2%) | 951 (12.0%) | 455 (10.7%) | 189 (7.9%) | 171 (6.6%) | 3945 (19.7%) | 782 (13.8%) | 3046 (18.2%) | 1716 (14.4%) | 758 (10.7%) | 485 (12.0%) |
| 31-40 | 2945 (25.3%) | 748 (20.5%) | 2157 (27.3%) | 1221 (28.7%) | 616 (25.9%) | 634 (24.5%) | 5230 (26.1%) | 1357 (23.9%) | 4597 (27.5%) | 3351 (28.2%) | 1930 (27.2%) | 1040 (25.7%) |
| 41-50 | 3553 (30.5%) | 1138 (31.2%) | 2411 (30.5%) | 1360 (31.9%) | 800 (33.6%) | 894 (34.5%) | 5274 (26.3%) | 1592 (28.0%) | 4572 (27.3%) | 3548 (29.8%) | 2273 (32.0%) | 1208 (29.9%) |
| 51-64 | 3459 (29.7%) | 1498 (41.1%) | 2380 (30.1%) | 1224 (28.7%) | 774 (32.5%) | 894 (34.5%) | 5627 (28.0%) | 1951 (34.3%) | 4509 (27.0%) | 3275 (27.5%) | 2139 (30.1%) | 1306 (32.3%) |
| **Country of birth** |  |  |  |  |  |  |  |  |  |  |  |  |
| Sweden | 10122 (87.0%) | 3165 (86.8%) | 6896 (87.3%) | 3763 (88.3%) | 2099 (88.2%) | 2016 (77.7%) | 16878 (84.1%) | 4901 (86.3%) | 14321 (85.6%) | 10311 (86.7%) | 6180 (87.0%) | 3393 (84.0%) |
| Nordic countries | 355 (3.0%) | 122 (3.3%) | 222 (2.8%) | 127 (3.0%) | 65 (2.7%) | 65 (2.5%) | 431 (2.1%) | 112 (2.0%) | 308 (1.8%) | 217 (1.8%) | 141 (2.0%) | 84 (2.1%) |
| EU25 | 251 (2.2%) | 63 (1.7%) | 170 (2.2%) | 87 (2.0%) | 52 (2.2%) | 59 (2.3%) | 509 (2.5%) | 123 (2.2%) | 393 (2.4%) | 308 (2.6%) | 168 (2.4%) | 97 (2.4%) |
| Rest of the world | 913 (7.8%) | 295 (8.1%) | 611 (7.7%) | 283 (6.6%) | 163 (6.9%) | 453 (17.5%) | 2258 (11.2%) | 546 (9.6%) | 1702 (10.2%) | 1054 (8.9%) | 611 (8.6%) | 465 (11.5%) |
| **Level of education** |  |  |  |  |  |  |  |  |  |  |  |  |
| Elementary | 1073 (9.2%) | 387 (10.6%) | 719 (9.1%) | 321 (7.5%) | 238 (10.0%) | 351 (13.5%) | 1565 (7.8%) | 460 (8.1%) | 1257 (7.5%) | 800 (6.7%) | 474 (6.7%) | 428 (10.6%) |
| High school | 5516 (47.4%) | 1680 (46.1%) | 3644 (46.1%) | 1818 (42.7%) | 986 (41.4%) | 1284 (49.5%) | 9224 (45.9%) | 2561 (45.1%) | 7324 (43.8%) | 5010 (42.1%) | 2836 (39.9%) | 1891 (46.8%) |
| Collage, university | 5052 (43.4%) | 1578 (43.3%) | 3536 (44.8%) | 2121 (49.8%) | 1155 (48.5%) | 958 (36.9%) | 9287 (46.3%) | 2661 (46.8%) | 8143 (48.7%) | 6080 (51.1%) | 3790 (53.4%) | 1720 (42.6%) |
| **Type of living area** |  |  |  |  |  |  |  |  |  |  |  |  |
| Big city | 4852 (41.7%) | 1363 (37.4%) | 3380 (42.8%) | 1813 (42.6%) | 950 (39.9%) | 969 (37.4%) | 7340 (36.6%) | 2179 (38.3%) | 6174 (36.9%) | 4235 (35.6%) | 2418 (34.1%) | 1354 (33.5%) |
| Medium-sized city | 3953 (34.0%) | 1247 (34.2%) | 2591 (32.8%) | 1432 (33.6%) | 772 (32.5%) | 867 (33.4%) | 7917 (39.4%) | 2140 (37.7%) | 6450 (38.6%) | 4694 (39.5%) | 3037 (42.8%) | 1678 (41.5%) |
| Small city/village | 2836 (24.4%) | 1035 (28.4%) | 1928 (24.4%) | 1015 (23.8%) | 657 (27.6%) | 757 (29.2%) | 4819 (24.0%) | 1363 (24.0%) | 4100 (24.5%) | 2961 (24.9%) | 1645 (23.2%) | 1007 (24.9%) |
| **Family situation** |  |  |  |  |  |  |  |  |  |  |  |  |
| Married or cohabitant without children | 1610 (13.8%) | 636 (17.4%) | 1052 (13.3%) | 584 (13.7%) | 307 (12.9%) | 342 (13.2%) | 3488 (17.4%) | 1039 (18.3%) | 2782 (16.6%) | 2008 (16.9%) | 1259 (17.7%) | 638 (15.8%) |
| Married or cohabitant with children | 4902 (42.1%) | 1396 (38.3%) | 3369 (42.7%) | 1874 (44.0%) | 1067 (44.9%) | 974 (37.6%) | 6819 (34.0%) | 1876 (33.0%) | 5934 (35.5%) | 4529 (38.1%) | 2772 (39.0%) | 1313 (32.5%) |
| Single without children | 3621 (31.1%) | 1026 (28.1%) | 2355 (29.8%) | 1230 (28.9%) | 669 (28.1%) | 845 (32.6%) | 7920 (39.5%) | 2159 (38.0%) | 6311 (37.7%) | 4130 (34.7%) | 2289 (32.2%) | 1559 (38.6%) |
| Single with children | 1508 (13.0%) | 587 (16.1%) | 1123 (14.2%) | 572 (13.4%) | 336 (14.1%) | 432 (16.7%) | 1849 (9.2%) | 608 (10.7%) | 1697 (10.1%) | 1223 (10.3%) | 780 (11.0%) | 529 (13.1%) |
| **Occupational status** |  |  |  |  |  |  |  |  |  |  |  |  |
| White collar | 6011 (51.6%) | 1877 (51.5%) | 4167 (52.8%) | 2585 (60.7%) | 1473 (61.9%) | 1065 (41.1%) | 9793 (48.8%) | 2800 (49.3%) | 8756 (52.4%) | 6867 (57.8%) | 4279 (60.3%) | 1737 (43.0%) |
| Blue collar | 5296 (45.5%) | 1616 (44.3%) | 3539 (44.8%) | 1567 (36.8%) | 834 (35.1%) | 1223 (47.2%) | 8786 (43.8%) | 2404 (42.3%) | 6718 (40.2%) | 4116 (34.6%) | 2272 (32.0%) | 1604 (39.7%) |
| Missing information | 334 (2.9%) | 152 (4.2%) | 193 (2.4%) | 108 (2.5%) | 72 (3.0%) | 305 (11.8%) | 1497 (7.5%) | 478 (8.4%) | 1250 (7.5%) | 907 (7.6%) | 549 (7.7%) | 698 (17.3%) |

Distributions of working life characteristics in each trajectory group of monthly sickness absence and disability pension days among individuals who started a sickness absence spell due to stress-related disorders.

|  | Cohort-2011 | | | | | | Cohort-2018 | | | | | |
| --- | --- | --- | --- | --- | --- | --- | --- | --- | --- | --- | --- | --- |
| Name of trajectory groups | **Steep drop** | **Fluctuating** | **Fast decrease** | **Medium decrease** | **Slow decrease** | **Constant high** | **Steep drop** | **Constant fluctuating** | **Fast decrease** | **Medium decrease** | **Slow decrease** | **Constant high** |
| N of participants | 11,641 | 3645 | 7899 | 4260 | 2379 | 2593 | 20,076 | 5682 | 16,724 | 11,890 | 7100 | 4039 |
| Employment status at the start of the sickness absence spell | | | | | | | | | | | | |
| Employed/student | 11,145 (95.8%) | 3342 (91.7%) | 7577 (95.9%) | 4048 (95.0%) | 2227 (93.6%) | 1741 (67.2%) | 19,498 (97.1%) | 5454 (96.0%) | 16,224 (97.0%) | 11,531 (97.0%) | 6793 (95.7%) | 3305(81.8%) |
| Unemployed | 341 (2.9%) | 279 (7.7%) | 247 (3.1%) | 177 (4.2%) | 120 (5.0%) | 820 (31.6%) | 402 (2.0%) | 180 (3.2%) | 347 (2.1%) | 271 (2.3%) | 235(3.3%) | 636 (15.7%) |
| Parental leave | 148 (1.3%) | 24 (0.7%) | 75 (0.9%) | 35 (0.8%) | 32 (1.3%) | 32 (1.2%) | 52 (0.3%) | 10 (0.2%) | 50 (0.3%) | 29 (0.2%) | 17 (0.2%) | 25 (0.6%) |
| Unknown |  |  |  |  |  |  | 124 (0.6%) | 38 (0.7%) | 103 (0.6%) | 59 (0.5%) | 55 (0.8%) | 73 (1.8%) |
| Extent at the start of the sickness absence spell | | | | | | | | | | | | |
| 100% | 9551 (82.0%) | 1899 (52.1%) | 6517 (82.5%) | 3655 (85.8%) | 2078 (87.3%) | 2232 (86.1%) | 16,274 (81.1%) | 3515 (61.9%) | 13,300 (79.5%) | 10,410 (87.6%) | 6412(90.3%) | 3565 (88.3%) |
| 75% | 120 (1.0%) | 352 (9.7%) | 115 (1.5%) | 93 (2.2%) | 72 (3.0%) | 74 (2.9%) | 262 (1.3%) | 350(6.2%) | 307 (1.8%) | 240 (2.0%) | 180 (2.5%) | 106 (2.6%) |
| 50% | 1536 (13.2%) | 1084 (29.7%) | 932 (11.8%) | 429 (10.1%) | 200 (8.4%) | 252 (9.7%) | 2821 (14.1%) | 1456 (25.6%) | 2393 (14.3%) | 1066 (9.0%) | 434 (6.1%) | 305 (7.6%) |
| 25% | 434 (3.7%) | 310 (8.5%) | 335 (4.2%) | 83 (1.9%) | 29 (1.2%) | 35 (1.3%) | 719 (3.6%) | 361 (6.4%) | 724 (4.3%) | 174 (1.5%) | 74 (1.0%) | 63 (1.6%) |
| Sickness absence days due to stress-related diagnosis in the preceding year | | | | | | | | | | | | |
| 0 | 11,321 (97.2%) | 3362 (92.3%) | 7519 (95.2%) | 4054 (95.1%) | 2292 (96.3%) | 2464 (95.0%) | 19,461 (96.9%) | 5143 (90.5%) | 15,877 (94.9%) | 11,360 (95.6%) | 6770 (95.4%) | 3754 (93.0%) |
| 0.25 to 50 | 253 (2.2%) | 158 (4.3%) | 268 (3.4%) | 127 (3.0%) | 50 (2.1%) | 51 (2.0%) | 421 (2.1%) | 290 (5.1%) | 527 (3.2%) | 326 (2.7%) | 205 (2.9%) | 127 (3.1%) |
| 50.25 to 90 | 40 (0.3%) | 43 (1.2%) | 64 (0.8%) | 45 (1.1%) | 20 (0.8%) | 29 (1.1%) | 104 (0.5%) | 115 (2.0%) | 168 (1.0%) | 101 (0.8%) | 65 (0.9%) | 68 (1.7%) |
| 90.25 and over | 27 (0.2%) | 80 (2.2%) | 48 (0.6%) | 34 (0.8%) | 17 (0.7%) | 49 (1.9%) | 90 (0.4%) | 134 (2.4%) | 152 (0.9%) | 103 (0.9%) | 60 (0.8%) | 90 (2.2%) |
| Sickness absence days due to other mental diagnosis in the preceding year | | | | | | | | | | | | |
| 0 | 11,396 (97.9%) | 3388 (92.9%) | 7634 (96.6%) | 4111 (96.5%) | 2273 (95.5%) | 2390 (92.2%) | 19,760 (98.4%) | 5386 (94.8%) | 16,340 (97.7%) | 11,589 (97.5%) | 6889 (97.0%) | 3795 (94.0%) |
| 0.25 to 50 | 172 (1.5%) | 144 (4.0%) | 176 (2.2%) | 76 (1.8%) | 64 (2.7%) | 66 (2.5%) | 221 (1.1%) | 169 (3.0%) | 232 (1.4%) | 173 (1.5%) | 118 (1.7%) | 84 (2.1%) |
| 50.25 to 90 | 39 (0.3%) | 47 (1.3%) | 39 (0.5%) | 25 (0.6%) | 17 (0.7%) | 34 (1.3%) | 51 (0.3%) | 59 (1.0%) | 71 (0.4%) | 63 (0.5%) | 35 (0.5%) | 46 (1.1%) |
| 90.25 and over | 34 (0.3%) | 66 (1.8%) | 50 (0.6%) | 48 (1.1%) | 25 (1.1%) | 103 (4.0%) | 44 (0.3%) | 78 (1.2%) | 81 (0.4%) | 65 (0.6%) | 58 (0.8%) | 104 (2.8%) |
| Sickness absence days due to somatic diagnosis in the preceding year | | | | | | | | | | | | |
| 0 | 10,680 (91.7%) | 3026 (83.0%) | 6923 (87.6%) | 3765 (88.4%) | 2090 (87.9%) | 2226 (85.8%) | 19,370 (96.5%) | 5151 (90.7%) | 15,923 (95.2%) | 11,326 (95.3%) | 6683 (94.1%) | 3709 (91.8%) |
| 0.25 to 50 | 807 (6.9%) | 468 (12.8%) | 795 (10.1%) | 408 (9.6%) | 233 (9.8%) | 228 (8.8%) | 566 (2.8%) | 371 (6.5%) | 626 (3.7%) | 443 (3.7%) | 309 (4.4%) | 197 (4.9%) |
| 50.25 to 90 | 87 (0.7%) | 86 (2.4%) | 103 (1.3%) | 46 (1.1%) | 25 (1.1%) | 34 (1.3%) | 76 (0.4%) | 90 (1.6%) | 97 (0.6%) | 69 (0.6%) | 49 (0.7%) | 56 (1.4%) |
| 90.25 and over | 67 (0.6%) | 65 (1.7%) | 78 (1.0%) | 41 (1.0%) | 31 (1.3%) | 105 (3.1%) | 64 (0.4%) | 61 (1.2%) | 18 (0.5%) | 52 (0.5%) | 59 (0.5%) | 77 (1.9%) |
| Disability pension in the preceding year | | | | | | | | | | | | |
| None | 11,586 (99.5%) | 2654 (72.8%) | 7836 (99.2%) | 4197 (98.5%) | 2163 (90.9%) | 1898 (73.2%) | 20,051 (99.9%) | 5161 (90.8%) | 16,704 (99.9%) | 11,859 (99.7%) | 6811 (95.9%) | 3771 (93.4%) |
| Any | 55 (0.5%) | 991 (27.2%) | 63 (0.8%) | 63 (1.5%) | 216 (9.1%) | 695 (26.8%) | 25 (0.1%) | 521 (9.2%) | 20 (0.1%) | 31 (0.3%) | 289 (4.1%) | 268 (6.6%) |

Distributions of health care use in each trajectory group of monthly sickness absence and disability pension days among individuals who started a sickness absence spell due to stress-related disorders.

|  | Cohort-2011 | | | | | | Cohort-2018 | | | | | |
| --- | --- | --- | --- | --- | --- | --- | --- | --- | --- | --- | --- | --- |
|  | **Steep drop** | **Constant fluctuating** | **Fast decrease** | **Medium decrease** | **Slow decrease** | **Constant high** | **Steep drop** | **Constant fluctuating** | **Fast decrease** | **Medium decrease** | **Slow decrease** | **Constant high** |
| N of participants | 11,641 | 3645 | 7899 | 4260 | 2379 | 2593 | 20,076 | 5682 | 16,724 | 11,890 | 7100 | 4039 |
| Specialized outpatient healthcare visits with stress-related diagnosis in the preceding year | | | | | | | | | | | | |
| 0 | 11,472 (98.5%) | 3460 (94.9%) | 7717 (97.7%) | 4148 (97.4%) | 2285 (96.0%) | 2201 (84.9%) | 19,724 (98.2%) | 5377 (94.6%) | 16,267 (97.3%) | 11,529 (97.0%) | 6776 (95.4%) | 3577 (88.6%) |
| 1 | 122 (1.0%) | 92 (2.5%) | 133 (1.7%) | 66 (1.5%) | 51 (2.1%) | 158 (6.1%) | 227 (1.1%) | 140 (2.5%) | 239 (1.4%) | 177 (1.5%) | 158 (2.2%) | 167 (4.1%) |
| 2-3 | 34 (0.3%) | 60 (1.6%) | 38 (0.5%) | 27 (0.6%) | 27 (1.1%) | 136 (5.2%) | 85 (0.4%) | 93 (1.6%) | 143 (0.9%) | 125 (1.1%) | 107 (1.5%) | 176 (4.4%) |
| ≥4 | 13 (0.1%) | 33 (0.9%) | 11 (0.1%) | 19 (0.4%) | 16 (0.7%) | 98 (3.8%) | 40 (0.2%) | 72 (1.3%) | 75 (0.4%) | 59 (0.5%) | 59 (0.8%) | 119 (2.9%) |
| Specialized outpatient healthcare visits with other mental diagnosis in the preceding year | | | | | | | | | | | | |
| 0 | 11,295 (97.0%) | 3304 (90.6%) | 7564 (95.8%) | 4063 (95.4%) | 2235 (93.9%) | 2099 (80.9%) | 19,219 (95.7%) | 5096 (89.7%) | 15,832 (94.7%) | 11,251 (94.6%) | 6587 (92.8%) | 3386 (83.8%) |
| 1 | 211 (1.8%) | 159 (4.4%) | 179 (2.3%) | 104 (2.4%) | 52 (2.2%) | 183 (7.1%) | 472 (2.4%) | 256 (4.5%) | 461 (2.8%) | 308 (2.6%) | 240 (3.4%) | 230 (5.7%) |
| 2-3 | 98 (0.8%) | 102 (2.8%) | 88 (1.1%) | 53 (1.2%) | 43 (1.8%) | 181 (7.0%) | 250 (1.2%) | 179 (3.2%) | 279 (1.7%) | 202 (1.7%) | 164 (2.3%) | 220 (5.4%) |
| ≥4 | 37 (0.3%) | 80 (2.2%) | 68 (0.9%) | 40 (0.9%) | 49 (2.1%) | 130 (5.0%) | 135 (0.7%) | 151 (2.7%) | 152 (0.9%) | 129 (1.1%) | 109 (1.5%) | 203 (5.0%) |
| Specialized outpatient healthcare visits with somatic diagnosis in the preceding year | | | | | | | | | | | | |
| 0 | 7651 (65.7%) | 1883 (51.7%) | 4813 (60.9%) | 2558 (60.0%) | 1354 (56.9%) | 1405 (54.2%) | 12,346 (61.5%) | 2798 (49.2%) | 9598 (57.4%) | 6904 (58.1%) | 3821 (53.8%) | 1998 (49.5%) |
| 1 | 2108 (18.1%) | 754 (20.7%) | 1461 (18.5%) | 834 (19.6%) | 494 (20.8%) | 519 (20.0%) | 3710 (18.5%) | 1158 (20.4%) | 3166 (18.9%) | 2221 (18.7%) | 1424 (20.1%) | 821 (20.3%) |
| 2-3 | 1308 (11.2%) | 605 (16.6%) | 1071 (13.6%) | 598 (14.0%) | 360 (15.1%) | 414 (16.0%) | 2659 (13.2%) | 990 (17.4%) | 2484 (14.9%) | 1768 (14.9%) | 1155 (16.3%) | 680 (16.8%) |
| ≥4 | 574 (4.9%) | 403 (11.1%) | 554 (7.0%) | 270 (6.3%) | 171 (7.2%) | 255 (9.8%) | 1361 (6.8%) | 736 (13.0%) | 1476 (8.8%) | 997 (8.4%) | 700 (9.9%) | 540 (13.4%) |
| Specialized outpatient healthcare visits with any diagnosis in the preceding year | | | | | | | | | | | | |
| 0 | 7416 (63.7%) | 1682 (46.1%) | 4598 (58.2%) | 2444 (57.4%) | 1261 (53.0%) | 1106 (42.7%) | 11,889 (59.2%) | 2548 (44.8%) | 9142 (54.7%) | 6563 (55.2%) | 3553 (50.0%) | 1689 (41.8%) |
| 1 | 2168 (18.6%) | 759 (20.8%) | 1498 (19.0%) | 845 (19.8%) | 489 (20.6%) | 495 (19.1%) | 3772 (18.8%) | 1126 (19.8%) | 3212 (19.2%) | 2251 (18.9%) | 1442 (20.3%) | 773 (19.1%) |
| 2-3 | 1393 (12.0%) | 670 (18.4%) | 1153 (14.6%) | 630 (14.8%) | 385 (16.2%) | 500 (19.3%) | 2829 (14.1%) | 1061 (18.7%) | 2608 (15.6%) | 1866 (15.7%) | 1219 (17.2%) | 772 (19.1%) |
| ≥4 | 664 (5.7%) | 534 (14.7%) | 650 (8.2%) | 341 (8.0%) | 244 (10.3%) | 492 (19.0%) | 1586 (7.9%) | 947 (16.7%) | 1762 (10.5%) | 1210 (10.2%) | 886 (12.5%) | 805 (19.9%) |
| Inpatient care days for stress-related diagnosis in the preceding year | | | | | | | | | | | | |
| No | 11,591 (99.6%) | 3618 (99.3%) | 7856 (99.5%) | 4233 (99.4%) | 2365 (99.4%) | 2534 (97.7%) | 20,009 (99.7%) | 5654 (99.5%) | 16,658 (99.6%) | 11,843 (99.6%) | 7060 (99.4%) | 3982 (98.6%) |
| Yes | 50 (0.4%) | 27 (0.7%) | 43 (0.5%) | 27 (0.6%) | 14 (0.6%) | 59 (2.3%) | 67 (0.3%) | 28 (0.5%) | 66 (0.4%) | 47 (0.4%) | 40 (0.6%) | 57 (1.4%) |
| Inpatient care days for other mental diagnosis in the preceding year | | | | | | | | | | | | |
| No | 11,575 (99.4%) | 3590 (98.5%) | 7810 (98.9%) | 4217 (99.0%) | 2348 (98.7%) | 2490 (96.0%) | 19,956 (99.4%) | 5593 (98.4%) | 16,579 (99.1%) | 11,803 (99.3%) | 7023 (98.9%) | 3923 (97.1%) |
| Yes | 66 (0.6%) | 55 (1.5%) | 89 (1.1%) | 43 (1.0%) | 31 (1.3%) | 103 (4.0%) | 120 (0.6%) | 89 (1.6%) | 145 (0.9%) | 87 (0.7%) | 77 (1.1%) | 116 (2.9%) |
| Inpatient care days for somatic diagnosis in the preceding year | | | | | | | | | | | | |
| No | 10,917 (93.8%) | 3264 (89.5%) | 7239 (91.6%) | 3924 (92.1%) | 2193 (92.2%) | 2297 (88.6%) | 19,130 (95.3%) | 5237 (92.2%) | 15,718 (94.0%) | 11,234 (94.5%) | 6652 (93.7%) | 3682 (91.2%) |
| Yes | 724 (6.2%) | 381 (10.5%) | 660 (8.4%) | 336 (7.9%) | 186 (7.8%) | 296 (11.4%) | 946 (4.7%) | 445 (7.8%) | 1006 (6.0%) | 656 (5.5%) | 448 (6.3%) | 357 (8.8%) |
| Inpatient care days for any diagnosis in the preceding year | | | | | | | | | | | | |
| No | 10,858 (93.3%) | 3231 (88.6%) | 7176 (90.8%) | 3888 (91.3%) | 2169 (91.2%) | 2234 (86.2%) | 19,047 (94.9%) | 5197 (91.5%) | 15,614 (93.4%) | 11,176 (94.0%) | 6597 (92.9%) | 3601 (89.2%) |
| Yes | 783 (6.7%) | 414 (11.4%) | 723 (9.2%) | 372 (8.7%) | 210 (8.8%) | 359 (13.8%) | 1029 (5.1%) | 485 (8.5%) | 1110 (6.6%) | 714 (6.0%) | 503 (7.1%) | 438 (10.8%) |
