# Supplementary material for: Sickness absence and disability pension trajectories among individuals on sickness absence due to stress-related disorders. Two prospective population-based cohorts with 13-month follow-up
Source: PLoS One. 2024 Dec 13;19(12):e0315706. doi: 10.1371/journal.pone.0315706 (PMC11643301; doi:10.1371/journal.pone.0315706)
Supplement: S2 Table — (DOCX) [file pone.0315706.s007.docx]

**Supplement Table 2.**

Associations between trajectory group membership and sociodemographic, work and morbidity related variables, estimated using a multinomial logistic regression, amongst individuals with sickness absence (SA) due to stress-related disorders in **2011**.

| **Characteristics (Cohort 2011)** | **Constant fluctuating** | | **Fast decrease** | | **Medium decrease** | | **Slow decrease** | | **Constant high** | |
| --- | --- | --- | --- | --- | --- | --- | --- | --- | --- | --- |
|  | **OR** | **95% CI** | **OR** | **95% CI** | **OR** | **95% CI** | **OR** | **95% CI** | **OR** | **95% CI** |
| **Sex** |  |  |  |  |  |  |  |  |  |  |
| Women | Reference | Reference | Reference | Reference | Reference | Reference | Reference | Reference | Reference | Reference |
| Men | 0.73 | 0.66 – 0.81 | 0.96 | 0.89 – 1.02 | 0.94 | 0.86 – 1.02 | 0.80 | 0.71 – 0.89 | 0.82 | 0.73 – 0.92 |
| **Age (years)** |  |  |  |  |  |  |  |  |  |  |
| 18 to 30 | Reference | Reference | Reference | Reference | Reference | Reference | Reference | Reference | Reference | Refereme |
| 31 to 40 | 1.46 | 1.23 – 1.72 | 1.25 | 1.12 – 1.38 | 1.36 | 1.20 – 1.56 | 1.66 | 1.38 – 2.00 | 2.14 | 1.75 – 2.62 |
| 41 to 50 | 1.52 | 1.29 – 1.79 | 1.14 | 1.02 – 1.26 | 1.23 | 1.08 – 1.41 | 1.69 | 1.40 – 2.03 | 2.19 | 1.79 – 2.68 |
| 51 to 64 | 1.57 | 1.34 – 1.86 | 1.17 | 1.05 – 1.30 | 1.12 | 0.98 – 1.28 | 1.64 | 1.36 – 1.97 | 2.27 | 1.85 – 2.78 |
| **Country of birth** |  |  |  |  |  |  |  |  |  |  |
| Sweden | Reference | Reference | Reference | Reference | Reference | Reference | Reference | Reference | Reference | Reference |
| Other Nordic countries | 0.89 | 0.70 – 1.13 | 0.89 | 0.75 – 1.06 | 0.95 | 0.77 – 1.17 | 0.80 | 0.61 – 1.05 | 0.79 | 0.58 – 1.06 |
| Other EU countries | 0.74 | 0.54 – 1.00 | 0.97 | 0.79 – 1.18 | 0.89 | 0.70 – 1.15 | 0.95 | 0.70 – 1.30 | 0.95 | 0.68 – 1.33 |
| Other countries | 1.06 | 0.91 – 1.24 | 0.95 | 0.85 – 1.06 | 0.84 | 0.72 – 0.96 | 0.88 | 0.73 – 1.05 | 1.58 | 1.35 – 1.85 |
| **Education level** |  |  |  |  |  |  |  |  |  |  |
| Elementary | Reference | Reference | Reference | Reference | Reference | Reference | Reference | Reference | Reference | Reference |
| High school | 0.92 | 0.79 – 1.06 | 0.99 | 0.89 – 1.10 | 1.07 | 0.94 – 1.23 | 0.78 | 0.67 – 0.92 | 0.94 | 0.80 – 1.11 |
| Collage, university | 0.98 | 0.84 – 1.16 | 1.05 | 0.93 – 1.17 | 1.18 | 1.01 – 1.37 | 0.82 | 0.69 – 0.98 | 1.01 | 0.84 – 1.21 |
| **Type of living area** |  |  |  |  |  |  |  |  |  |  |
| Big city | Reference | Reference | Reference | Reference | Reference | Reference | Reference | Reference | Reference | Reference |
| Medium-sized city | 1.02 | 0.93 – 1.13 | 0.94 | 0.88 – 1.00 | 0.98 | 0.90 – 1.07 | 1.00 | 0.90 – 1.11 | 1.03 | 0.91 – 1.15 |
| Small city/village | 1.19 | 1.07 – 1.32 | 0.98 | 0.91 – 1.06 | 1.00 | 0.91 – 1.10 | 1.24 | 1.11 – 1.39 | 1.30 | 1.15 – 1.47 |
| **Family situation** |  |  |  |  |  |  |  |  |  |  |
| Married or cohabitant without children | Reference | Reference | Reference | Reference | Reference | Reference | Reference | Reference | Reference | Reference |
| Married or cohabitant with children | 1.07 | 0.93 – 1.22 | 1.08 | 0.98 – 1.19 | 1.03 | 0.91 – 1.16 | 1.33 | 1.14 – 1.55 | 1.19 | 1.01 – 1.40 |
| Single without children | 1.00 | 0.87 – 1.15 | 1.04 | 0.94 – 1.15 | 0.96 | 0.85 – 1.09 | 1.20 | 1.03 – 1.41 | 1.25 | 1.06 – 1.48 |
| Single with children | 1.23 | 1.05 – 1.44 | 1.13 | 1.00 – 1.27 | 1.00 | 0.87 – 1.16 | 1.26 | 1.05 – 1.52 | 1.27 | 1.05 – 1.54 |
| **Occupational status** |  |  |  |  |  |  |  |  |  |  |
| White collar | Reference | Reference | Reference | Reference | Reference | Reference | Reference | Reference | Reference | Reference |
| Blue collar | 0.98 | 0.88 – 1.08 | 0.98 | 0.91 – 1.05 | 0.69 | 0.63 – 0.75 | 0.57 | 0.50 – 0.64 | 0.90 | 0.79 – 1.01 |
| Missing information | 1.46 | 1.16 – 1.84 | 0.90 | 0.74 – 1.08 | 0.80 | 0.63 – 1.00 | 0.81 | 0.61 – 1.07 | 1.96 | 1.55 – 2.47 |
| **Employment status at the start of the SA spell** | | | | | | | | | | |
| Employed/student | Reference | Reference | Reference | Reference | Reference | Reference | Reference | Reference | Reference | Reference |
| Unemployed | 1.59 | 1.32 – 1.92 | 0.97 | 0.82 – 1.15 | 1.31 | 1.08 – 1.59 | 1.26 | 1.01 – 1.58 | 5.67 | 4.83 – 6.66 |
| Parental leave/homemaker | 0.49 | 0.31 – 0.78 | 0.62 | 0.46 – 0.82 | 0.53 | 0.36 – 0.77 | 0.94 | 0.63 – 1.40 | 1.03 | 0.67 – 1.59 |
| **Extent at the start of the SA spell** | | | | | | | | | | |
| 100% | Reference | Reference | Reference | Reference | Reference | Reference | Reference | Reference | Reference | Reference |
| 75% | 3.90 | 3.02 – 5.04 | 1.30 | 1.00 – 1.69 | 1.54 | 1.17 – 2.05 | 0.81 | 0.58 – 1.14 | 0.35 | 0.24 – 0.50 |
| 50% | 1.74 | 1.56 – 1.93 | 0.83 | 0.76 – 0.90 | 0.60 | 0.53 – 0.68 | 0.31 | 0.26 – 0.37 | 0.19 | 0.16 – 0.23 |
| 25% | 1.61 | 1.35 – 1.94 | 0.98 | 0.85 – 1.14 | 0.37 | 0.29 – 0.47 | 0.14 | 0.09 – 0.21 | 0.10 | 0.07 – 0.15 |
| **SA days due to stress-related diagnosis in the preceding year** | | | | | | | | | | |
| 0 | Reference | Reference | Reference | Reference | Reference | Reference | Reference | Reference | Reference | Reference |
| 0.25 to 50 | 1.86 | 1.49 – 2.32 | 1.58 | 1.32 – 1.88 | 1.44 | 1.16 – 1.80 | 1.00 | 0.73 – 1.37 | 1.03 | 0.74 – 1.43 |
| 50.25 to 90 | 3.78 | 2.42 – 5.90 | 2.41 | 1.62 – 3.59 | 3.47 | 2.25 – 5.34 | 2.99 | 1.73 – 5.19 | 3.91 | 2.30 – 6.67 |
| 90.25 and over | 11.6 | 7.56 – 17.9 | 3.39 | 2.16 – 5.32 | 5.23 | 3.26 – 8.40 | 6.79 | 4.00 – 11.5 | 19.9 | 12.7 – 31.2 |
| **SA days due to other mental diagnosis in the preceding year** | | | | | | | | | | |
| 0 | Reference | Reference | Reference | Reference | Reference | Reference | Reference | Reference | Reference | Reference |
| 0.25 to 50 | 2.23 | 1.74 – 2.86 | 1.45 | 1.17 – 1.79 | 1.22 | 0.93 – 1.61 | 1.80 | 1.33 – 2.43 | 1.53 | 1.12 – 2.10 |
| 50.25 to 90 | 3.03 | 1.91 – 4.80 | 1.35 | 0.86 – 2.12 | 1.75 | 1.05 – 2.93 | 2.12 | 1.18 – 3.81 | 3.07 | 1.84 – 5.14 |
| 90.25 and over | 5.41 | 3.51 – 8.34 | 2.06 | 1.33 – 3.21 | 3.75 | 2.39 – 5.90 | 3.47 | 2.03 – 5.95 | 8.42 | 5.45 – 13.0 |
| **SA days due to somatic diagnosis in the preceding year** | | | | | | | | | | |
| 0 | Reference | Reference | Reference | Reference | Reference | Reference | Reference | Reference | Reference | Reference |
| 0.25 to 50 | 1.64 | 1.43 – 1.89 | 1.42 | 1.27 – 1.58 | 1.43 | 1.25 – 1.63 | 1.42 | 1.20 – 1.67 | 1.54 | 1.30 – 1.83 |
| 50.25 to 90 | 2.78 | 1.99 – 3.87 | 1.64 | 1.22 – 2.19 | 1.53 | 1.06 – 2.20 | 1.40 | 0.89 – 2.22 | 1.78 | 1.15 – 2.74 |
| 90.25 and over | 2.99 | 2.08 – 4.30 | 1.64 | 1.17 – 2.29 | 1.86 | 1.25 – 2.78 | 2.60 | 1.67 – 4.04 | 7.28 | 5.14 – 10.3 |
| **Disability pension in the preceding year** | | | | | | | | | | |
| None | Reference | Reference | Reference | Reference | Reference | Reference | Reference | Reference | Reference | Reference |
| Any | 40.3 | 30.3 – 53.7 | 1.72 | 1.19 – 2.49 | 3.89 | 2.67 – 5.67 | 36.6 | 26.5 – 50.6 | 116 | 84.5 – 158 |
| **Specialized outpatient healthcare visits with stress-related diagnosis in the preceding year** | | | | | | | | | | |
| 0 | Reference | Reference | Reference | Reference | Reference | Reference | Reference | Reference | Reference | Reference |
| 1 | 1.24 | 0.91 – 1.69 | 1.42 | 1.10 – 1.82 | 1.30 | 0.95 – 1.77 | 1.66 | 1.17 – 2.35 | 2.05 | 1.52 – 2.76 |
| 2-3 | 1.68 | 1.04 – 2.70 | 1.27 | 0.79 – 2.05 | 1.61 | 0.95 – 2.73 | 2.24 | 1.30 – 3.87 | 2.67 | 1.68 – 4.25 |
| ≥4 | 1.70 | 0.84 – 3.44 | 0.80 | 0.35 – 1.82 | 2.37 | 1.14 – 4.95 | 2.29 | 1.03 – 5.06 | 3.60 | 1.82 – 7.12 |
| **Specialized outpatient healthcare visits with other mental diagnosis in the preceding year** | | | | | | | | | | |
| 0 | Reference | Reference | Reference | Reference | Reference | Reference | Reference | Reference | Reference | Reference |
| 1 | 1.62 | 1.27 – 2.06 | 1.11 | 0.90 – 1.36 | 1.21 | 0.95 – 1.55 | 0.99 | 0.72 – 1.37 | 2.10 | 1.64 – 2.70 |
| 2-3 | 2.10 | 1.52 – 2.89 | 1.16 | 0.86 – 1.56 | 1.28 | 0.90 – 1.81 | 1.57 | 1.07 – 2.30 | 3.33 | 2.44 – 4.56 |
| ≥4 | 3.32 | 2.14 – 5.14 | 2.10 | 1.38 – 3.17 | 2.09 | 1.30 – 3.34 | 3.94 | 2.47 – 6.27 | 3.93 | 2.54 – 6.10 |
| **Specialized outpatient healthcare visits with somatic diagnosis in the preceding year** | | | | | | | | | | |
| 0 | Reference | Reference | Reference | Reference | Reference | Reference | Reference | Reference | Reference | Reference |
| 1 | 1.29 | 1.16 – 1.44 | 1.07 | 1.0 – 1.16 | 1.17 | 1.07 – 1.29 | 1.30 | 1.16 – 1.46 | 1.16 | 1.02 – 1.31 |
| 2-3 | 1.44 | 1.27 – 1.62 | 1.20 | 1.10 – 1.32 | 1.28 | 1.15 – 1.43 | 1.43 | 1.25 – 1.64 | 1.25 | 1.08 – 1.44 |
| ≥4 | 1.81 | 1.54 – 2.13 | 1.30 | 1.14 – 1.48 | 1.20 | 1.02 – 1.41 | 1.39 | 1.14 – 1.69 | 1.43 | 1.18 – 1.74 |
| **Inpatient care days for stress-related diagnosis in the preceding year** | | | | | | | | | | |
| No | Reference | Reference | Reference | Reference | Reference | Reference | Reference | Reference | Reference | Reference |
| Yes | 1.18 | 0.70 – 2.00 | 0.92 | 0.60 – 1.41 | 1.08 | 0.66 – 1.78 | 0.86 | 0.45 – 1.62 | 1.33 | 0.81 – 2.19 |
| **Inpatient care days for other mental diagnosis in the preceding year** | | | | | | | | | | |
| No | Reference | Reference | Reference | Reference | Reference | Reference | Reference | Reference | Reference | Reference |
| Yes | 1.09 | 0.72 – 1.67 | 1.44 | 1.02 – 2.03 | 1.24 | 0.82 – 1.88 | 1.35 | 0.84 – 2.18 | 1.59 | 1.06 – 2.37 |
| **Inpatient care days for somatic diagnosis in the preceding year** | | | | | | | | | | |
| No | Reference | Reference | Reference | Reference | Reference | Reference | Reference | Reference | Reference | Reference |
| Yes | 1.14 | 0.97 – 1.33 | 1.15 | 1.02 – 1.29 | 1.09 | 0.94 – 1.27 | 0.97 | 0.80 – 1.16 | 1.10 | 0.92 – 1.32 |

OR: odds ratio, CI: confidence interval

Associations between trajectory group membership and sociodemographic, work and healthcare related variables, estimated using a multinomial logistic regression, amongst individuals with sickness absence (SA) due to stress-related disorders in **2018.**

| **Characteristic (Cohort 2018)** | **Constant fluctuating** | | **Fast decrease** | | **Medium decrease** | | **Slow decrease** | | **Constant high** | |
| --- | --- | --- | --- | --- | --- | --- | --- | --- | --- | --- |
|  | **OR** | **95% CI** | **OR** | **95% CI** | **OR** | **95% CI** | **OR** | **95% CI** | **OR** | **95% CI** |
| **Sex** |  |  |  |  |  |  |  |  |  |  |
| Women | Reference | Reference | Reference | Reference | Reference | Reference | Reference | Reference | Reference | Reference |
| Men | 0.75 | 0.69 – 0.81 | 0.91 | 0.87 – 0.96 | 0.90 | 0.85 – 0.95 | 0.78 | 0.73 – 0.83 | 0.76 | 0.69 – 0.83 |
| **Age (years)** |  |  |  |  |  |  |  |  |  |  |
| 18 to 30 | Reference | Reference | Reference | Reference | Reference | Reference | Reference | Reference | Reference | Reference |
| 31 to 40 | 1.25 | 1.12 – 1.39 | 1.09 | 1.02 – 1.16 | 1.34 | 1.24 – 1.44 | 1.71 | 1.55 – 1.89 | 1.84 | 1.61 – 2.09 |
| 41 to 50 | 1.40 | 1.26 – 1.55 | 1.06 | 0.99 – 1.13 | 1.37 | 1.26 – 1.48 | 1.94 | 1.75 – 2.15 | 2.22 | 1.96 – 2.53 |
| 51 to 64 | 1.53 | 1.38 – 1.70 | 1.01 | 0.94 – 1.08 | 1.26 | 1.17 – 1.37 | 1.82 | 1.64 – 2.01 | 2.36 | 2.08 – 2.69 |
| **Country of birth** |  |  |  |  |  |  |  |  |  |  |
| Sweden | Reference | Reference | Reference | Reference | Reference | Reference | Reference | Reference | Reference | Reference |
| Other Nordic countries | 0.79 | 0.64 – 0.99 | 0.85 | 0.73 – 0.99 | 0.81 | 0.69 – 0.96 | 0.83 | 0.68 – 1.01 | 0.83 | 0.65 – 1.07 |
| Other EU countries | 0.90 | 0.73 – 1.10 | 0.92 | 0.80 – 1.05 | 0.96 | 0.83 – 1.12 | 0.85 | 0.71 – 1.02 | 0.89 | 0.71 – 1.13 |
| Other countries | 0.86 | 0.77 – 0.95 | 0.90 | 0.84 – 0.96 | 0.76 | 0.70 – 0.82 | 0.70 | 0.63 – 0.77 | 0.85 | 0.76 – 0.96 |
| **Education level** |  |  |  |  |  |  |  |  |  |  |
| Elementary | Reference | Reference | Reference | Reference | Reference | Reference | Reference | Reference | Reference | Reference |
| High school | 0.94 | 0.83 – 1.05 | 0.96 | 0.89 – 1.05 | 1.02 | 0.93 – 1.12 | 0.99 | 0.88 – 1.11 | 0.86 | 0.76 – 0.98 |
| Collage, university | 0.95 | 0.84 – 1.08 | 0.98 | 0.90 – 1.07 | 1.02 | 0.92 – 1.12 | 1.06 | 0.94 – 1.19 | 0.84 | 0.73 – 0.96 |
| **Type of living area** |  |  |  |  |  |  |  |  |  |  |
| Big city | Reference | Reference | Reference | Reference | Reference | Reference | Reference | Reference | Reference | Reference |
| Medium-sized city | 0.92 | 0.86 – 0.99 | 0.95 | 0.91 – 1.00 | 0.99 | 0.94 – 1.05 | 1.12 | 1.06 – 1.20 | 1.10 | 1.02 – 1.20 |
| Small city/village | 0.93 | 0.86 – 1.01 | 1.02 | 0.97 – 1.08 | 1.06 | 1.00 – 1.13 | 1.01 | 0.94 – 1.09 | 1.05 | 0.96 – 1.16 |
| **Family situation** |  |  |  |  |  |  |  |  |  |  |
| Married or cohabitant without children | Reference | Reference | Reference | Reference | Reference | Reference | Reference | Reference | Reference | Reference |
| Married or cohabitant with children | 1.23 | 1.11 – 1.36 | 1.06 | 0.99 – 1.14 | 1.13 | 1.05 – 1.23 | 1.24 | 1.13 – 1.36 | 1.32 | 1.17 – 1.50 |
| Single without children | 1.12 | 1.02 – 1.24 | 1.01 | 0.94 – 1.08 | 0.99 | 0.92 – 1.06 | 0.98 | 0.89 – 1.07 | 1.23 | 1.10 – 1.38 |
| Single with children | 1.25 | 1.09 – 1.42 | 1.09 | 1.00 – 1.20 | 1.13 | 1.02 – 1.25 | 1.22 | 1.08 – 1.37 | 1.50 | 1.29 – 1.73 |
| **Occupational status** |  |  |  |  |  |  |  |  |  |  |
| White collar | Reference | Reference | Reference | Reference | Reference | Reference | Reference | Reference | Reference | Reference |
| Blue collar | 1.01 | 0.94 – 1.10 | 0.87 | 0.82 – 0.91 | 0.66 | 0.62 – 0.70 | 0.60 | 0.56 – 0.64 | 0.89 | 0.81 – 0.97 |
| Missing information | 1.14 | 1.01 – 1.28 | 0.94 | 0.87 – 1.03 | 0.87 | 0.79 – 0.96 | 0.83 | 0.75 – 0.93 | 1.62 | 1.44 – 1.83 |
| **Employment status at the start of the SA spell** | | | | | | | | | | |
| Employed/student | Reference | Reference | Reference | Reference | Reference | Reference | Reference | Reference | Reference | Reference |
| Unemployed | 1.14 | 0.95 – 1.38 | 0.98 | 0.84 – 1.13 | 1.05 | 0.90 – 1.23 | 1.40 | 1.18 – 1.65 | 5.03 | 4.36 – 5.81 |
| Parental leave/homemaker | 0.54 | 0.27 – 1.08 | 0.96 | 0.65 – 1.42 | 0.79 | 0.50 – 1.26 | 0.75 | 0.43 – 1.32 | 1.91 | 1.14 – 3.20 |
| Unknown | 0.75 | 0.51 – 1.09 | 0.85 | 0.65 – 1.11 | 0.68 | 0.49 – 0.93 | 0.91 | 0.65 – 1.28 | 1.70 | 1.22 – 2.37 |
| **Extent at the start of the SA spell** | | | | | | | | | | |
| 100% | Reference | Reference | Reference | Reference | Reference | Reference | Reference | Reference | Reference | Reference |
| 75% | 3.01 | 2.50 – 3.62 | 1.35 | 1.14 – 1.60 | 1.23 | 1.03 – 1.47 | 0.62 | 0.50 – 0.78 | 0.53 | 0.41 – 0.70 |
| 50% | 1.64 | 1.51 – 1.77 | 0.97 | 0.91 – 1.03 | 0.51 | 0.47 – 0.55 | 0.22 | 0.20 – 0.25 | 0.24 | 0.21 – 0.28 |
| 25% | 1.43 | 1.24 – 1.65 | 1.06 | 0.95 – 1.18 | 0.30 | 0.25 – 0.35 | 0.15 | 0.12 – 0.19 | 0.20 | 0.15 – 0.27 |
| **SA days due to stress-related diagnosis in the preceding year** | | | | | | | | | | |
| 0 | Reference | Reference | Reference | Reference | Reference | Reference | Reference | Reference | Reference | Reference |
| 0.25 to 50 | 2.34 | 1.99 – 2.74 | 1.49 | 1.31 – 1.70 | 1.38 | 1.19 – 1.60 | 1.45 | 1.22 – 1.73 | 1.67 | 1.35 – 2.06 |
| 50.25 to 90 | 3.75 | 2.85 – 4.93 | 1.91 | 1.49 – 2.45 | 1.87 | 1.41 – 2.47 | 2.14 | 1.56 – 2.95 | 3.75 | 2.70 – 5.20 |
| 90.25 and over | 4.34 | 3.40 – 5.54 | 1.80 | 1.42 – 2.27 | 2.13 | 1.65 – 2.75 | 2.41 | 1.80 – 3.21 | 5.65 | 4.33 – 7.37 |
| **SA days due to other mental diagnosis in the preceding year** | | | | | | | | | | |
| 0 | Reference | Reference | Reference | Reference | Reference | Reference | Reference | Reference | Reference | Reference |
| 0.25 to 50 | 2.39 | 1.93 – 2.95 | 1.23 | 1.02 – 1.48 | 1.42 | 1.16 – 1.74 | 1.63 | 1.29 – 2.06 | 1.77 | 1.35 – 2.31 |
| 50.25 to 90 | 3.56 | 2.42 – 5.24 | 1.59 | 1.11 – 2.29 | 2.21 | 1.52 – 3.21 | 2.05 | 1.32 – 3.19 | 3.40 | 2.21 – 5.23 |
| 90.25 and over | 4.09 | 2.77 – 6.06 | 2.01 | 1.39 – 2.92 | 2.52 | 1.70 – 3.72 | 3.70 | 2.46 – 5.56 | 6.94 | 4.73 – 10.2 |
| **SA days due to somatic diagnosis in the preceding year** | | | | | | | | | | |
| 0 | Reference | Reference | Reference | Reference | Reference | Reference | Reference | Reference | Reference | Reference |
| 0.25 to 50 | 2.03 | 1.77 – 2.35 | 1.27 | 1.13 – 1.43 | 1.37 | 1.20 – 1.56 | 1.56 | 1.35 – 1.81 | 1.73 | 1.46 – 2.07 |
| 50.25 to 90 | 3.37 | 2.45 – 4.63 | 1.42 | 1.05 – 1.93 | 1.60 | 1.15 – 2.23 | 1.78 | 1.23 – 2.57 | 3.18 | 2.21 – 4.59 |
| 90.25 and over | 2.95 | 2.08 – 4.19 | 1.34 | 0.96 – 1.87 | 1.43 | 0.99 – 2.08 | 2.57 | 1.78 – 3.71 | 4.27 | 2.98 – 6.11 |
| **Disability pension in the preceding year** | | | | | | | | | | |
| None | Reference | Reference | Reference | Reference | Reference | Reference | Reference | Reference | Reference | Reference |
| Any | 44.0 | 29.1 – 66.4 | 0.97 | 0.54 – 1.74 | 3.09 | 1.81 – 5.27 | 90.2 | 58.9 – 138 | 108 | 69.5 – 166 |
| **Specialized outpatient healthcare visits with stress-related diagnosis in the preceding year** | | | | | | | | | | |
| 0 | Reference | Reference | Reference | Reference | Reference | Reference | Reference | Reference | Reference | Reference |
| 1 | 1.38 | 1.09 – 1.73 | 1.14 | 0.94 – 1.37 | 1.27 | 1.03 – 1.55 | 1.74 | 1.40 – 2.16 | 2.02 | 1.61 – 2.54 |
| 2-3 | 1.87 | 1.36 – 2.57 | 1.68 | 1.27 – 2.22 | 2.24 | 1.68 – 2.99 | 2.74 | 2.02 – 3.73 | 3.82 | 2.84 – 5.15 |
| ≥4 | 2.01 | 1.32 – 3.05 | 1.65 | 1.11 – 2.47 | 1.85 | 1.21 – 2.82 | 2.55 | 1.65 – 3.94 | 3.12 | 2.06 – 4.71 |
| **Specialized outpatient healthcare visits with other mental diagnosis in the preceding year** | | | | | | | | | | |
| 0 | Reference | Reference | Reference | Reference | Reference | Reference | Reference | Reference | Reference | Reference |
| 1 | 1.57 | 1.33 – 1.86 | 1.10 | 0.96 – 1.26 | 1.08 | 0.93 – 1.25 | 1.30 | 1.10 – 1.54 | 1.71 | 1.43 – 2.05 |
| 2-3 | 1.72 | 1.39 – 2.13 | 1.16 | 0.97 – 1.39 | 1.21 | 1.0 – 1.47 | 1.43 | 1.15 – 1.78 | 2.05 | 1.65 – 2.55 |
| ≥4 | 2.05 | 1.57 – 2.69 | 1.06 | 0.82 – 1.35 | 1.31 | 1.01 – 1.70 | 1.46 | 1.10 – 1.95 | 2.62 | 1.99 – 3.43 |
| **Specialized outpatient healthcare visits with somatic diagnosis in the preceding year** | | | | | | | | | | |
| 0 | Reference | Reference | Reference | Reference | Reference | Reference | Reference | Reference | Reference | Reference |
| 1 | 1.30 | 1.20 – 1.41 | 1.08 | 1.02 – 1.14 | 1.06 | 1.00 – 1.13 | 1.20 | 1.12 – 1.30 | 1.24 | 1.13 – 1.36 |
| 2-3 | 1.38 | 1.26 – 1.50 | 1.16 | 1.09 – 1.23 | 1.17 | 1.09 – 1.25 | 1.30 | 1.20 – 1.42 | 1.27 | 1.14 – 1.41 |
| ≥4 | 1.78 | 1.59 – 1.99 | 1.30 | 1.20 – 1.41 | 1.23 | 1.13 – 1.35 | 1.40 | 1.26 – 1.56 | 1.55 | 1.37 – 1.76 |
| **Inpatient care days for stress-related diagnosis in the preceding year** | | | | | | | | | | |
| No | Reference | Reference | Reference | Reference | Reference | Reference | Reference | Reference | Reference | Reference |
| Yes | 0.70 | 0.43 – 1.14 | 0.91 | 0.63 – 1.30 | 0.93 | 0.63 – 1.39 | 0.95 | 0.62 – 1.46 | 1.02 | 0.67 – 1.57 |
| **Inpatient care days for other mental diagnosis in the preceding year** | | | | | | | | | | |
| No | Reference | Reference | Reference | Reference | Reference | Reference | Reference | Reference | Reference | Reference |
| Yes | 1.25 | 0.91 – 1.71 | 1.20 | 0.92 – 1.56 | 1.00 | 0.74 – 1.35 | 1.18 | 0.86 – 1.63 | 1.26 | 0.92 – 1.73 |
| **Inpatient care days for somatic diagnosis in the preceding year** | | | | | | | | | | |
| No | Reference | Reference | Reference | Reference | Reference | Reference | Reference | Reference | Reference | Reference |
| Yes | 1.14 | 1.00 – 1.30 | 1.11 | 1.01 – 1.23 | 1.06 | 0.95 – 1.18 | 1.07 | 0.94 – 1.22 | 1.14 | 0.98 – 1.33 |
