# Supplementary material for: Sickness absence and disability pension trajectories among individuals on sickness absence due to stress-related disorders. Two prospective population-based cohorts with 13-month follow-up
Source: PLoS One. 2024 Dec 13;19(12):e0315706. doi: 10.1371/journal.pone.0315706 (PMC11643301; doi:10.1371/journal.pone.0315706)
Supplement: S3 Table — (DOCX) [file pone.0315706.s008.docx]

S3 Table. Statistical characteristics of alternative trajectory models for the 2011 and 2018 cohort.

| **2011 cohort** |  |  |  |  |  |  |  |  |  |  |
| --- | --- | --- | --- | --- | --- | --- | --- | --- | --- | --- |
| **Number of trajectory groups** | **Degree of trajectory polynomial** | **Mean APPA** | **Min APPA** | **Entropy** | **Relative Entropy** | **Log-Likelihood** | **BIC** | **AIC** | **MSE** | **RMSE** |
| 2 | 3 | 0,990 | 0,987 | 658,6 | 0,971 | -601835 | -601887 | -601845 | 55,5 | 7,4 |
| 3 | 3 | 0,982 | 0,972 | 1277,5 | 0,964 | -568934 | -569012 | -568949 | 41,4 | 6,4 |
| 4 | 3 | 0,972 | 0,944 | 2020,2 | 0,955 | -549861 | -549965 | -549881 | 35,2 | 5,9 |
| 5 | 3 | 0,966 | 0,947 | 2330,3 | 0,955 | -539225 | -539355 | -539250 | 29,8 | 5,5 |
| 6 | 3 | 0,948 | 0,916 | 3818,9 | 0,934 | -534533 | -534688 | -534563 | 27,7 | 5,3 |
| 7 | 3 | 0,955 | 0,918 | 2790,4 | 0,956 | -530010 | -530191 | -530045 | 27,5 | 5,2 |
| 8 | 3 | 0,942 | 0,885 | 3413,1 | 0,949 | -526199 | -526407 | -526239 | 25,3 | 5,0 |
| 9 | 3 | 0,945 | 0,904 | 4275,0 | 0,940 | -523754 | -523988 | -523799 | 24,7 | 5,0 |
|  |  |  |  |  |  |  |  |  |  |  |
| **Number of trajectory groups** | **Degree of trajectory polynomial** | **Mean APPA** | **Min APPA** | **Entropy** | **Relative Entropy** | **Log-Likelihood** | **BIC** | **AIC** | **MSE** | **RMSE** |
| 6 | 2 | 0,952 | 0,904 | 3403,0 | 0,849 | -537155 | -537279 | -537179 | 29,5 | 5,4 |
| 6 | 3 | 0,948 | 0,916 | 3818,9 | 0,893 | -534533 | -534688 | -534563 | 27,7 | 5,3 |
| 6 | 4 | 0,950 | 0,906 | 3926,4 | 0,913 | -533244 | -533431 | -533280 | 26,9 | 5,2 |
|  |  |  |  |  |  |  |  |  |  |  |
|  |  |  |  |  |  |  |  |  |  |  |
|  |  |  |  |  |  |  |  |  |  |  |
| **2018 cohort** |  |  |  |  |  |  |  |  |  |  |
| **Number of trajectory groups** | **Degree of trajectory polynomial** | **Mean APPA** | **Min APPA** | **Entropy** | **Relative Entropy** | **Log-Likelihood** | **BIC** | **AIC** | **MSE** | **RMSE** |
| 2 | 3 | 0,985 | 0,980 | 2117,7 | 0,953 | -1297303 | -1297359 | -1297313 | 57,8 | 7,6 |
| 3 | 3 | 0,972 | 0,957 | 4325,9 | 0,940 | -1234445 | -1234528 | -1234460 | 42,1 | 6,5 |
| 4 | 3 | 0,966 | 0,948 | 5176,9 | 0,943 | -1199591 | -1199702 | -1199611 | 37,0 | 6,1 |
| 5 | 3 | 0,959 | 0,944 | 5831,7 | 0,945 | -1175701 | -1175840 | -1175726 | 31,4 | 5,6 |
| 6 | 3 | 0,947 | 0,923 | 7912,3 | 0,933 | -1164465 | -1164632 | -1164495 | 28,8 | 5,4 |
| 7 | 3 | 0,949 | 0,925 | 7561,1 | 0,941 | -1151802 | -1151996 | -1151837 | 28,0 | 5,3 |
| 8 | 3 | 0,942 | 0,914 | 9164,8 | 0,933 | -1150524 | -1150745 | -1150564 | 27,6 | 5,3 |
| 9 | 3 | 0,935 | 0,902 | 9710,6 | 0,933 | -1144307 | -1144557 | -1144352 | 26,2 | 5,1 |
|  |  |  |  |  |  |  |  |  |  |  |
| **Number of trajectory groups** | **Degree of trajectory polynomial** | **Mean APPA** | **Min APPA** | **Entropy** | **Relative Entropy** | **Log-Likelihood** | **BIC** | **AIC** | **MSE** | **RMSE** |
| 6 | 2 | 0,943 | 0,912 | 9906,8 | 0,782 | -1166966 | -1167099 | -1166990 | 29,5 | 5,4 |
| 6 | 3 | 0,947 | 0,923 | 7912,3 | 0,890 | -1164465 | -1164632 | -1164495 | 28,8 | 5,4 |
| 6 | 4 | 0,947 | 0,925 | 7863,3 | 0,913 | -1163968 | -1164168 | -1164004 | 28,9 | 5,4 |

Mean APPA: Mean Average Posterior Probability of Assignment

Min APPA: Minimum Average Posterior Probability of Assignment

Entropy

Relative Entropy

loglik: Log-Likelihood

BIC: Bayesian Information Criterion

AIC: Akaike Information Criterion

MSE: Mean Squared Error

RMSE: Root Mean Squared Error
