# Supplementary material for: Sickness absence and disability pension trajectories among individuals on sickness absence due to stress-related disorders. Two prospective population-based cohorts with 13-month follow-up
Source: PLoS One. 2024 Dec 13;19(12):e0315706. doi: 10.1371/journal.pone.0315706 (PMC11643301; doi:10.1371/journal.pone.0315706)
Supplement: S4 Table — (DOCX) [file pone.0315706.s009.docx]

**Supplement Table 4.**

The goodness of fit measures, comparing multinomial logistic regressions (fully adjusted and with one covariate excluded at a time) modeling the associations between sociodemographic, work, and health-related variables with trajectory group members, amongst individuals with sickness absence due to stress-related disorders in **2011.**

| **Cohort 2011** | Likelihood ratio test comparing full vs reduced model | | | Nagelkerke pseudo R^2^ Comparing full vs. reduced model |
| --- | --- | --- | --- | --- |
| **Covariate** | Chisquare | Degrees of freedom | p-value |  |
| Sex | 47.423 | 5 | <0.001 | 0.113 |
| Age | 137.270 | 15 | <0.001 | 0.329 |
| Country of birth | 64.864 | 15 | <0.001 | 0.155 |
| Level of education | 18.746 | 10 | 0.044 | 0.045 |
| Type of living area | 43.251 | 10 | <0.001 | 0.103 |
| Family situation | 33.540 | 15 | 0.004 | 0.080 |
| Occupational status | 210.796 | 10 | <0.001 | 0.505 |
| Employment status at the start of the sickness absence spell | 595.687 | 10 | <0.001 | 1.437 |
| Extent at the start of the sickness absence spell | 1295.025 | 15 | <0.001 | 3.158 |
| Sickness absence days due to stress-related diagnosis in the preceding year | 369.360 | 15 | <0.001 | 0.888 |
| Sickness absence days due to other mental diagnosis in the preceding year | 193.326 | 15 | <0.001 | 0.463 |
| Sickness absence days due to somatic diagnosis in the preceding year | 226.094 | 15 | <0.001 | 0.542 |
| Disability pension in the preceding year | 2743.621 | 5 | <0.001 | 6.842 |
| Specialized outpatient healthcare visits with stress-related diagnosis in the preceding year | 67.554 | 15 | <0.001 | 0.162 |
| Specialized outpatient healthcare visits with other mental diagnosis in the preceding year | 141.712 | 15 | <0.001 | 0.339 |
| Specialized outpatient healthcare visits with somatic diagnosis in the preceding year | 109.120 | 15 | <0.001 | 0.261 |
| Inpatient care days for stress-related diagnosis in the preceding year | 2.891 | 5 | 0.717 | 0.007 |
| Inpatient care days for other mental diagnosis in the preceding year | 8.148 | 5 | 0.148 | 0.019 |
| Inpatient care days for somatic diagnosis in the preceding year | 7.575 | 5 | 0.181 | 0.018 |

The goodness of fit measures, comparing multinomial logistic regressions (fully adjusted and with one covariate excluded at a time) modeling the associations between sociodemographic, work, and health-related variables with trajectory group members, amongst individuals with sickness absence due to stress-related disorders in **2018.**

| **Cohort 2018** | Likelihood ratio test comparing full vs reduced model | | | Nagelkerke pseudo R^2^ Comparing full vs. reduced model |
| --- | --- | --- | --- | --- |
| **Covariate** | Chisquare | Degree of freedom | p-value |  |
| Sex | 104.181 | 5 | <0.001 | 0.142 |
| Age | 431.953 | 15 | <0.001 | 0.590 |
| Country of birth | 90.258 | 15 | <0.001 | 0.123 |
| Level of education | 14.039 | 10 | 0.171 | 0.019 |
| Type of living area | 54.288 | 10 | <0.001 | 0.074 |
| Family situation | 89.929 | 15 | <0.001 | 0.122 |
| Occupational status | 436.991 | 10 | <0.001 | 0.596 |
| Employment status at the start of the sickness absence spell | 671.226 | 15 | <0.001 | 0.918 |
| Extent at the start of the sickness absence spell | 2543.373 | 15 | <0.001 | 3.528 |
| Sickness absence days due to stress-related diagnosis in the preceding year | 426.768 | 15 | <0.001 | 0.582 |
| Sickness absence days due to other mental diagnosis in the preceding year | 242.223 | 15 | <0.001 | 0.330 |
| Sickness absence days due to somatic diagnosis in the preceding year | 255.330 | 15 | <0.001 | 0.348 |
| Disability pension in the preceding year | 2176.726 | 5 | <0.001 | 3.011 |
| Specialized outpatient healthcare visits with stress-related diagnosis in the preceding year | 150.447 | 15 | <0.001 | 0.205 |
| Specialized outpatient healthcare visits with other mental diagnosis in the preceding year | 140.444 | 15 | <0.001 | 0.191 |
| Specialized outpatient healthcare visits with somatic diagnosis in the preceding year | 188.812 | 15 | <0.001 | 0.257 |
| Inpatient care days for stress-related diagnosis in the preceding year | 2.748 | 5 | 0.739 | 0.004 |
| Inpatient care days for other mental diagnosis in the preceding year | 4.359 | 5 | 0.499 | 0.006 |
| Inpatient care days for somatic diagnosis in the preceding year | 6.631 | 5 | 0.250 | 0.009 |
